# Regulation of Neuroinflammation through Programed Death-1/Programed Death Ligand Signaling in Neurological Disorders

**DOI:** 10.3389/fncel.2014.00271

**Published:** 2014-09-03

**Authors:** Shangfeng Zhao, Fengwu Li, Rehana K. Leak, Jun Chen, Xiaoming Hu

**Affiliations:** ^1^Department of Neurology, University of Pittsburgh School of Medicine, Pittsburgh, PA, USA; ^2^Department of Neurosurgery, Beijing Tongren Hospital, Capital Medical University, Beijing, China; ^3^Institute of Neuroscience, Luhe Hospital, Capital Medical University, Beijing, China; ^4^Division of Pharmaceutical Sciences, Duquesne University, Pittsburgh, PA, USA

**Keywords:** PD-1, PD-L1, stroke, neurodegeneration, inflammation

## Abstract

Immune responses in the central nervous system (CNS), which involve both resident glial cells and infiltrating peripheral immune cells, play critical roles in the progress of brain injuries and neurodegeneration. To avoid inflammatory damage to the compromised brain, the immune cell activities in the CNS are controlled by a plethora of chemical mediators and signal transduction cascades, such as inhibitory signaling through programed death-1 (PD-1) and programed death ligand (PD-L) interactions. An increasing number of recent studies have highlighted the importance of PD-1/PD-L pathway in immune regulation in CNS disorders such as ischemic stroke, multiple sclerosis, and Alzheimer’s disease. Here, we review the current knowledge of the impact of PD-1/PD-L signaling on brain injury and neurodegeneration. An improved understanding of the function of PD-1/PD-L in the cross-talk between peripheral immune cells, CNS glial cells, and non-immune CNS cells is expected to shed further light on immunomodulation and help develop effective and safe immunotherapies for CNS disorders.

## Introduction

The central nervous system (CNS) was traditionally thought to tolerate the invasion of antigens without an inflammatory response. The presence of an intact blood–brain barrier (BBB) and the lack of lymphatic vessels in the brain was believed to restrict the infiltration of peripheral immune cells into brain parenchyma under physiological conditions and maintain the CNS in a so-called “immune-privileged” state (Engelhardt, 2008). Recent research, however, has resulted in a revision of this concept. Compelling data now suggest that the CNS is actually immunocompetent and not completely immune-privileged. A series of neuroinflammatory responses, involving both resident CNS glial cells and peripheral immune cells invading via the damaged BBB, are promptly launched in response to noxious stimuli (Zipp and Aktas, 2006). These immune cells are important for defense against CNS infection, injury, or neurodegeneration and for CNS repair and regeneration. Their activities, however, have to be carefully regulated to avoid inflammatory damage to already compromised and highly vulnerable tissues of the CNS.

The tendency of the immune system to damage bystander tissue is kept in check by a series of self-regulating, inhibitory systems that preserve immune homeostasis. For example, inhibitory signaling through programed death-1 (PD-1) and programed death ligand (PD-L) interactions is an important mechanism underlying immune regulation in many pathological circumstances, such as autoimmune diseases, cancer, and organ transplantation. Recent evidence indicates that the PD-1/PD-L system is also critical in reducing the inflammatory responses in CNS diseases such as stroke, multiple sclerosis (MS), and Alzheimer’s disease (AD) (Kroner et al., 2005; Ren et al., 2011b; Saresella et al., 2012). Here, we review our accumulated understanding of the PD-1/PD-L pathway, with a special emphasis on its potential role in brain injuries and neurodegenerative diseases.

## Functions of PD-1/PD-L1 in Immune Responses

Programed death-1 (or CD279) is a 50–55 kDa member of the CD28 family of T-cell regulators (Riley and June, 2005). It is expressed at a low level on naïve T-cells and can be induced upon activation in many types of immune cells, including T-cells, B cells, natural killer (NK) cells, monocytes, and dendritic cells (DCs). Structurally, PD-1 is composed of an N-terminal IgV-like domain, an approximately 20 amino acid-long stalk, a transmembrane domain, and a cytoplasmic domain. The cytoplasmic domain contains two tyrosine-based signaling motifs: an immunoreceptor tyrosine-based inhibitory motif (ITIM) and an immunoreceptor tyrosine-based switch motif (ITSM), both of which are essential for PD-1 function (Zhang et al., 2004). PD-1 has two binding ligands: PD-L1 (CD274) and PD-L2 (CD273). Both of these ligands are members of the B7 family of costimulatory molecules. PD-L1 is broadly expressed on a variety of hematopoietic cells (including T-cells, B cells, DCs, and monocytes) in addition to many non-hematopoietic cells, such as epithelial and endothelial cells. In contrast, the expression of PD-L2 is mainly restricted to antigen presenting cells (APCs), including macrophages, DCs, and specific B cell subpopulations (Zhong et al., 2007). In general, PD-L2 is expressed at lower levels but binds to PD-1 with higher affinity than PD-L1 (Ghiotto et al., 2010).

The breadth of expression of PD-1 and PD-L in multiple types of immune cells suggests a wide range of functions in immunomodulation. First and foremost, the main role of PD-1/PD-L1 is to act as a negative regulatory system to fine-tune T-cell and B cell activity. The engagement of T or B cell-expressed PD-1 with PD-L on APCs relays inhibitory signals that down-regulate T-cell receptor (TCR) or B cell receptor (BCR)-mediated cell activation (Freeman et al., 2000; Latchman et al., 2001; Okazaki et al., 2001; Yokosuka et al., 2012). As a consequence of this interaction and downstream effect, the PD-1/PD-L system plays critical roles in many T or B cell-mediated immune responses, including immunity to infection, antibody production, immune tolerance, and autoimmunity (Okazaki et al., 2013). For example, mounting evidence reveals the importance of the PD-1/PD-L pathway in the maintenance of central and peripheral tolerance. On the one hand, PD-1/PD-L1 interactions regulate T-cell selection and shape T-cell repertoires in the thymus (Blank et al., 2003; Keir et al., 2005). Absence of PD-1 alters the signaling threshold during T-cell development in thymus and leads to increased emergence of CD4/CD8 double-negative αβ T-cells. On the other hand, the PD-1/PD-L1 pathway also induces T-cell tolerance and inhibits self-reactive T-cell proliferation and cytokine production in peripheral lymph organs or tissues (Probst et al., 2005). Deficiency of PD-1 or blockade of PD-1/PD-L signaling results in the development or exacerbation of autoimmune diseases in mouse models of lupus-like glomerulonephritis/arthritis, cardiomyopathy, type I diabetes, experimental autoimmune encephalomyelitis (EAE), and autoimmune enteritis (Nishimura et al., 1999, 2001; Salama et al., 2003; Fife et al., 2009; Reynoso et al., 2009). These findings suggest that PD-1/PD-L signaling plays an important protective role against multiple types of autoimmune disorders. Interestingly, there is some variation in the autoimmune disease phenotype depending on the genetic background of different mouse stains, indicating perhaps that lymphocyte regulation through PD-1/PD-L is highly antigen specific (Okazaki et al., 2013).

Recent studies reveal that the PD-1/PD-L interaction also regulates the functions of cells other than lymphocytes through multiple mechanisms. First, the PD-1/PD-L1 interaction between T-cells and APCs may be bidirectional, enabling some degree of reciprocal communication. For example, PD-1 on T-cells activates PD-L1 on macrophages and induces a regulatory macrophage profile with enhanced IL-10 and reduced IL-6 production (Lee et al., 2013). Second, PD-1 signaling may function in APCs independently of TCR or BCR activation. One example is that the ligation of monocyte PD-1 with PD-L1 directly stimulates IL-10 production, leading to reversible CD4^+^ T-cell dysfunction after HIV infection (Said et al., 2010). In addition, cross-linking of PD-L2 on DCs with specific IgM directly stimulates DC functions and activates groups of genes involved in cell migration and survival (Blocki et al., 2006). These studies demonstrate that PD-1/PD-L signaling blunts immune overreaction and prevents cellular toxicity.

Unfortunately, our current knowledge about PD-1/PD-L functions in different immune cells is still limited. Further exploration of this field is expected to extend our understanding of the impact of this inhibitory signaling system and evaluate its therapeutic potential in immune-related diseases.

## PD-1/PD-L1 Signaling Pathway

PD-1/PD-L1 signaling has been studied most extensively in T and B lymphocytes. In these cells, PD-1 ligation induces signal transduction only when there is simultaneous activation of BCR or TCR. The binding of PD-1 with PD-L1, along with antigen recognition, results in the phosphorylation of tyrosine residues in the ITSM and subsequent recruitment of SH2 domain-containing phosphatase-2 (SHP-2), or less frequently, SHP-1 (Freeman et al., 2000; Latchman et al., 2001; Okazaki et al., 2001; Yokosuka et al., 2012). SHP-2 and SHP-1 are two highly related tyrosine phosphatases that dephosphorylate proximal signaling molecules such as Syk downstream of BCR or Zap70 downstream of TCR (Okazaki et al., 2001; Sheppard et al., 2004). This dephosphorylation attenuates the signaling cascades engaged by antigen recognition and diminishes the ensuing biological effects. The PD-1/PD-L1 activated signaling pathways in other types of cells remain to be characterized.

## PD-1 and PD-L1 in Ischemic Stroke

Stroke is an acute brain injury closely associated with strong and persistent inflammation. Post-stroke inflammation is characterized by the activation of local microglia and the rapid accumulation of peripheral immune cells in the ischemic brain (Iadecola and Anrather, 2011). The antigen-non-specific immune responses mediated by innate immune cells (microglia, macrophage, neutrophil, NK cells, etc.) commence very early after stroke. In contrast, the lymphocyte-mediated adaptive immune responses become prominent at later stages (since 3–4 days after onset) of stroke, although these lymphocytes may migrate into the ischemic boundary within the first 24 h of reperfusion (Gelderblom et al., 2009). Mounting evidence demonstrates that lymphocytes play pivotal roles in both brain injury and brain recovery. For instance, deficiency of either CD4^+^ or CD8^+^ T-cells resulted in the reduction in infarct volume and improvement in neurological performance in experimental models of stroke, suggesting detrimental roles of CD4^+^ and CD8^+^ T-cells after stroke (Yilmaz et al., 2006; Liesz et al., 2011). However, some specific lymphocyte populations, including regulating T-cells and regulating B cells, have been shown to be protective to ischemic brain or promote brain recovery after stroke (Liesz et al., 2009; Li et al., 2013). Further elucidating the mechanisms underlying intricate immunoregulation after stroke is critical not only for basic research of the immune system and CNS injury but also for the clinical translation of new therapeutic candidates.

**Figure 1:**
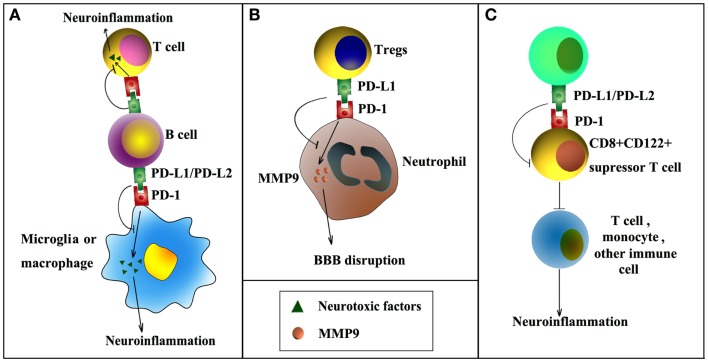
**PD-1/PD-L signaling in ischemic stroke**. PD-1/PD-L signaling may influence post-stroke inflammation and functional outcomes by negatively regulating the following cell–cell interactions. **(A)** PD-L1/PD-L2 expression on B cells inhibits the activation of microglia, macrophages, or effector T-cells, thereby reducing inflammation in the ischemic brain. **(B)** PD-L1 expression on regulatory T-cells (Tregs) inhibits neutrophil-derived matrix metalloproteinase-9 (MMP-9) through PD-L1–PD-1 interactions and reduces subsequent blood–brain barrier (BBB) damage in the acute phase after stroke. **(C)** PD-L1/PD-L2 inhibits immunoregulatory CD8^+^CD122^+^ suppressor T-cells, reducing their recruitment into the CNS from the spleen after stroke. As a result, post-stroke inflammation and brain injury are enhanced.

Several recent publications have highlighted the importance of PD-1 and PD-L1 signaling in post-stroke inflammation and brain injury (Figure 1). For example, it has been reported that the expression of PD-L1 and PD-L2 on peripheral B cells is significantly increased 4 days after transient middle cerebral artery occlusion (MCAO), an established experimental model of stroke (Ren et al., 2011b). In the meantime, the expression of PD-1 is elevated on activated resident microglia and on infiltrating macrophages. These elevations in PD-1/PD-L support the notion that this co-inhibitory pathway is intimately involved in the regulation of ischemic brain injury. Interestingly, experiments using PD-1 or PD-L1 knockout mice have shown diametrically opposed results. As would be expected from a protective role for PD-1, deficiency in PD-1 enlarges brain infarct sizes and exacerbates neurological deficits at 4 days after MCAO, and these events are accompanied by increased infiltration of CD3^+^ T-cells, Gr1^+^ neutrophils, macrophages, and exaggerated microglial activation (Ren et al., 2011b). In contrast, another study from the same research group using PD-L1 or PD-L2 knockout mice revealed that PD-L exacerbates post-stroke inflammation and plays a detrimental role in stroke outcomes (Bodhankar et al., 2013b). Mechanistically, the protective effects of PD-1 are attributed to its expression on B cells and subsequent inhibition of inflammatory responses in other immune effector cells (Ren et al., 2011b). The detrimental effects of PD-L1, however, may depend on its inhibition of the recruitment of immunoregulatory CD8^+^CD122^+^ suppressor T-cells from the spleen into the ischemic brain (Bodhankar et al., 2013b). CD8^+^CD122^+^ regulatory T-cells are known to regulate other CD8^+^CD122^−^ T-cells, which cause tissue damage when over-activated (Rifa’i et al., 2004). Thus, PD-L1 may release CD8^+^CD122^−^ T-cells from inhibition and thereby elicit injury. The opposing nature of PD-1 and PD-L in these studies may reflect the frequently dualistic nature of the immune system. Future immunotherapies will therefore have to account for the general complexity of immunomodulation in the injured brain.

A potential caveat of research on PD-1/PD-L is worth discussing here. All the above-mentioned studies on PD-1 and PD-L in stroke rely on global gene knockout mice. Although these results provide valuable information about the overall effects of PD-1 and PD-L on stroke outcome, they are by themselves not sufficient to define the cell-specific functions of PD-1 and PD-L in stroke. In this regard, further studies using transgenic mice with cell-specific gene manipulations are necessary. *In vitro* studies are also warranted to confirm direct cell–cell interactions. One recent study from our group demonstrated direct interactions between regulatory T-cells (Tregs) and neutrophils through PD-L1 and PD-1 (Li et al., 2014). This interaction was found to be essential for Treg-mediated suppression of neutrophil-derived matrix metalloproteinase-9 (MMP-9). In view of the importance of MMP-9 in early BBB disruption after stroke (Asahi et al., 2001; Rosell et al., 2006), we further showed that PD-L1 expression on Tregs mediates Treg-afforded neuroprotection against experimental stroke through the inhibition of peripheral neutrophil-derived MMP-9 and through subsequent preservation of BBB integrity (Li et al., 2014). In addition, another study demonstrated that adoptive transfer of IL-10-producing B cells into the stroke mice could increase the expression of PD-1 in peripheral CD4^+^ T-cells (Bodhankar et al., 2013a), suggesting that PD-1/PD-L1 interaction might also be important in regulatory B cell-provided neuroprotection after stroke (Ren et al., 2011a; Bodhankar et al., 2013a).

Thus far, all the research on PD-1/PD-L in stroke has focused on their short-term effects. Given the persistent immune responses after stroke and their contribution to brain recovery, the long-term influence of these inhibitory molecules in the ischemic brain is an important direction to pursue in the future.

## PD-1/PD-L1 Pathway in Neurodegenerative Diseases

### Alzheimer’s disease

Alzheimer’s disease is an age-related neurodegenerative disease characterized by memory loss, progressive cognitive impairment, and neuropsychiatric disturbances. The pathological hallmarks of AD are the extracellular accumulation of amyloid plaques and intracellular deposition of neurofibrillary tangles (Liu et al., 2013). Aβ accumulation in amyloid plaques leads to chronic neuroinflammation in the brain, thereby contributing to disease progression and poor functional outcomes (Rubio-Perez and Morillas-Ruiz, 2012). A reduction in suppressor cell function in the periphery has been observed in AD patients, as manifested by loss of balance in immune cell populations and decreased IL-10 production in the blood (Guerreiro et al., 2007; Speciale et al., 2007). Therefore, activation of immunoregulatory mechanisms during the progression of AD might be able to re-establish immune homeostasis.

Expression of PD-1 on CD4^+^ T-cells and PD-L1 on CD14^+^ monocyte/macrophage significantly decrease in AD patients and patients with mild cognitive impairment (MCI), underscoring the importance of these molecules in AD (Saresella et al., 2012). Impairments in PD-1/PD-L1 are associated with inhibition of IL-10 production, suggesting an effect of this signaling system in boosting IL-10 production. IL-10 has been shown to limit inflammatory responses and ameliorate AD pathology in animal models (Koronyo-Hamaoui et al., 2009). A recent study showed that although the IL-10 serum levels are comparable in AD patients and healthy controls, the frequency of CD4^+^ T-cells expressing IL-10 in AD group is much higher than that in controls, indication a systemic effort to counterbalance the pro-inflammatory responses in the AD brain (Torres et al., 2013). Thus, it is conceivable that a decrease in this protective cytokine in AD patients synergizes with an increased activity in Aβ-reactive T-cells, thereby enhancing neuroinflammation and exacerbating brain pathology. In addition, the PD-1/PD-L1 interaction is shown to induce the apoptosis in Aβ-specific CD4^+^ T-cells (Saresella et al., 2012).

The expression of PD-1 on Tregs is also affected by AD pathology (Saresella et al., 2010). The number of PD-1^+^ Tregs is increased both in patients with fully developed AD and with MCI. In contrast, PD-1^−^ Tregs are significantly increased only in MCI patients, but not in full-blown AD patients. Although PD-1 has been known to promote Treg differentiation (Wang et al., 2010), the functional differences between PD-1^−^ Tregs and PD-1^+^ Tregs are not clear. Therefore, the significance of altered PD-1 expression on Tregs in AD and MCI patients awaits further investigation.

To date, our knowledge of the function of PD-1/PD-L1 in the pathology of AD is very limited. Further work is necessary to elucidate the cellular and molecular mechanisms of PD-1 or PD-L1 actions in AD, such as their contribution to immune cell cross-talk in the CNS.

### Multiple sclerosis

Multiple sclerosis is a chronic inflammatory neurodegenerative disease characterized by the demyelination of white matter and focal infiltration of immune cells in the CNS (Minagar et al., 2004; Compston and Coles, 2008). The CD4^+^ effector T-cells have long been considered as the most important infiltrating cells in MS. The involvement of other T-cell subtypes (interleukin-17-producing T-cells, CD8^+^ T-cells, Tregs, and γ/φ T-cells), APCs, and microglia has also been supported (Viglietta et al., 2004; Langrish et al., 2005; Tzartos et al., 2008).

Mounting evidence highlights the importance of PD-1 and PD-L in MS. The expression of PD-1 is significantly increased on myelin basic protein (MBP)-stimulated CD4^+^ and CD8^+^ T lymphocytes isolated from the peripheral blood of patients with stable MS compared to lymphocytes from patients with acute remissions and relapses. Correspondingly, PD-L1-expressing APCs are increased in stable MS patients. Up-regulation of PD-1/PD-L1 enhances the apoptosis of MBP-specific cells, which is associated with disease remission in MS patients (Trabattoni et al., 2009). Moreover, an intronic 7146G/A polymorphisms within the PD-1 gene, which result in reduced inhibitory function of PD-1 on cytokine production and T-cell activation, are associated with a progressive disease course in MS patients (Kroner et al., 2005). These findings demonstrate a potential role of PD-1/PD-L1 in slowing the progression of MS. Consistent with these human studies, animal experiments in the EAE model of MS have shown that genetic ablation or pharmacological blockade of PD-1 or PD-L1 enhances the activation and expansion of T-cells and aggravates pathological alterations in the CNS (Latchman et al., 2001; Salama et al., 2003). In contrast, PD-L2 knockout mice develop similar pathologies as wild-type mice with no significant difference in severity (Carter et al., 2007). Furthermore, PD-L2 on microglial and CNS infiltrating APCs has been shown to be less potent than PD-L1 in the regulation of cytokine (IFN-γ, IL-17, etc.) production and the activation of auto-reactive T-cells (Schreiner et al., 2008). A study of PD-L1 or PD-L2 blockade in several mouse strains further suggests that differential effects of these two PD-L isoforms on the susceptibility and progression of EAE may be attributed to differences in genetic background (Zhu et al., 2006).

Mechanistic studies suggest that PD-1/PD-L signaling actively modulates the onset and progressive course of MS via the regulation of various types of immune cells, such as effector T-cells, DCs, Tregs, and NK T-cells (Latchman et al., 2004; Chang et al., 2008; Schreiner et al., 2008; Brandl et al., 2010). These mechanisms have been recently reviewed elsewhere (Joller et al., 2012) and are therefore not discussed further here. Due to the importance of the PD-1/PD-L system in MS, therapeutic strategies targeting PD-1/PD-L1 interactions can be envisioned as an immunosuppressive treatment for MS patients. For example, estrogen has been shown to effectively protect against EAE through upregulating PD-L1 expression on B cells and increase the amount of IL-10-producing regulatory B cells (Bodhankar et al., 2011). It also induces B-cell-dependent up-regulation of PD-1 on CD4^+^Foxp3^+^ Tregs, which provide further protection against EAE (Bodhankar et al., 2012). IL-12, a cytokine mainly produced by APCs, is also shown to suppress the development of EAE through stimulating IFN-γ production in APCs and enhancing downstream PD-1/PD-L1 signaling (Cheng et al., 2007). Further studies are warranted to assess the effectiveness of PD-1/PD-L1 modulation as a therapeutic strategy in MS patients.

## Conclusion

The expression of PD-1 and PD-L on many immune and non-immune cells surely allows for multiple tiers of immunoregulation in the CNS and remains an active area of investigation. Increasing numbers of clinical and experimental studies have shed light on the critical role of the PD-1/PD-L1 system in the regulation of resident microglia in the CNS and peripheral immune cells after brain injury and neurodegeneration. However, given the complexity of inflammatory responses in the CNS, our current understanding of the function of PD-1/PD-L in the cross-talk between peripheral immune cells, CNS glial cells, and non-immune CNS cells still lies in its infancy. As an example, the expression of PD-L1/PD-L2 is up-regulated in inflamed endothelial cells, with an intention to inhibit T-cell transmigration through BBB (Pittet et al., 2011). In particular, the impaired expression of PD-L2 on endothelial cells may contribute to the cerebral inflammation in MS patients. The molecular mechanism underlying the PD-L2-afforded BBB resistance to T-cell infiltration, however, is not clear. Similarly, the expression of PD-L on astrocytes has been reported in a model of nerve injury (Lipp et al., 2007), however, whether and how astrocytic PD-L plays a role in restricting local inflammation in CNS has not been examined. It will also be important to determine whether modulation of PD-1/PD-L signaling pathway during CNS injury or neurodegeneration influence the balance between debris clearance, brain repair, and inflammatory damage. Further investigations of the PD-1/PD-L pathway in CNS disorders are warranted to improve our understanding of the mechanisms underlying immunomodulation and to develop effective and safe immunotherapies.

## Conflict of Interest Statement

The authors declare that the research was conducted in the absence of any commercial or financial relationships that could be construed as a potential conflict of interest.
